# Effectiveness and safety of moxibustion for alleviating symptoms of overactive bladder: A prospective, randomized controlled, crossover-design, pilot study: Erratum

**DOI:** 10.1097/MD.0000000000012952

**Published:** 2018-10-19

In the article, “Effectiveness and safety of moxibustion for alleviating symptoms of overactive bladder: A prospective, randomized controlled, crossover-design, pilot study”,^[1]^ which appeared in Volume 97, Issue 34 of *Medicine*, the headings of Table 1 were misleading. The corrected table appears below:

**Table 1:**
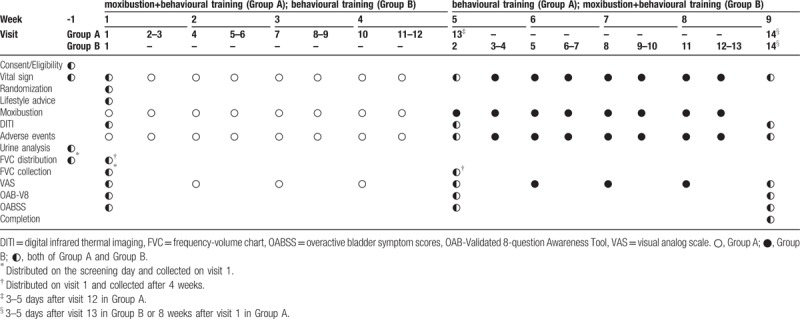
Schedule for intervention and outcome measurement.
